# Nonoperative Korean Medicine Combination Therapy for Lumbar Spinal Stenosis: A Retrospective Case-Series Study

**DOI:** 10.1155/2015/263898

**Published:** 2015-10-12

**Authors:** Kiok Kim, Yongjae Jeong, Yousuk Youn, Jeongcheol Choi, Jaehong Kim, Wonseok Chung, Tae-Hun Kim

**Affiliations:** ^1^Department of Spine Center, Mokhuri Neck & Back Hospital, Seoul 135-857, Republic of Korea; ^2^Department of Oriental Rehabilitation Medicine, College of Korean Medicine, Kyung Hee University, Seoul 130-872, Republic of Korea; ^3^Korean Medicine Clinical Trial Center, Korean Medicine Hospital, Kyung Hee University, Seoul, Republic of Korea

## Abstract

This is a retrospective case series exploring the therapeutic benefits and harm of nonoperative Korean medicine combination therapy for lumbar spinal stenosis (LSS). The medical records of a total of 33 LSS patients, who were treated as inpatients at Mokhuri Neck and Back Hospital, Republic of Korea, from November 2010 to January 2012, were reviewed first and telephone survey on these patients was conducted after one year. Body acupuncture, pharmacoacupuncture, Chuna, and oral administration of herbal medicines were offered to all patients. A Visual analogue scale (VAS) of pain and the walking duration without pain were used to assess the patients during the approximately 1-month treatment period. The average VAS score of pain and the walking duration improved significantly; the VAS score decreased from 9 (SD, 1.15) to 2.75 (2.22) (*p* < 0.01), and the walking duration increased from 5.5 (6.66) to 16.75 (13.00) minutes (*p* < 0.01). No adverse event was reported during the treatment. In addition, the decreased pain level and improved function continued for over one year. Although we did not find definitive evidence, the study results suggest that KM combination therapy may be beneficial for decreasing pain and improving function in LSS patients and may produce comparatively few adverse events.

## 1. Introduction

Lumbar spinal stenosis (LSS) refers to a narrowed spinal canal that commonly arises from degenerative changes in the bony structures or ligaments of the lumbar vertebrae. The characteristic symptoms of LSS include low back and leg pain in one or both sides and neurological claudication, which deteriorates in a lumbar extension posture [1]. Surgery to decompress the entrapped spinal cord is usually recommended, but complications occur quite often, and over 40% of patients reported complications after surgery in a previous report [2]. However, nonsurgical treatment has recently been identified as an important treatment strategy for decreasing pain and improving function. It is therefore commonly recommended as an initial therapy for LSS when no neurological contraindications or symptoms are evident [3].

Although a current Cochrane review on the effect of nonoperative treatment modalities for LSS only suggests a low quality of evidence [4], many procedures are currently used in clinical practice, for example, oral administration of analgesics including gabapentin or methylcobalamin, epidural steroid injections, home or intensive inpatient physical therapies, manual therapies, and multimodal nonsurgical therapies [5]. In Korean medicine (KM), several different nonsurgical interventions are currently used for the treatment of LSS. According to the clinical guidelines of the Korean Acupuncture and Moxibustion Medicine Society, acupuncture is recommended for the treatment of LSS. Korean pharmacoacupuncture, a comparatively novel intervention that consists of acupuncture-point injection of distillates of medicinal herbs, is frequently used for low back pain [6]. Various herbal decoctions are used for low back pain as single interventions or combined with acupuncture or other nondrug treatments [7, 8]. Because low back pain is the leading cause for visits to KM hospitals [9], clinical evidence regarding the various interventions of KM for spinal stenosis should be established. Observational studies including case studies and case series cannot provide conclusive evidence, but they play a complementary role to randomized controlled trials (RCTs). If no RCTs are available, case series can provide the best information with which practitioners and policy makers can make health care decisions [10]. The objective of this retrospective case series was to summarize the results of KM treatment of LSS in a local Korean hospital and provide basic evidence supporting its use and a clear outline of the proper procedures.

## 2. Methods

This was a retrospective case-series study. The study consisted of two parts; the medical records during the admission treatment period from all of the eligible patients were reviewed to assess the short-term effect of KM treatment for LSS first, and then a telephone follow-up survey of the available patients was conducted after one year to assess the midterm effects. The medical records of all patients who were diagnosed with LSS and treated as inpatients at the Mokhuri Neck and Back Hospital in the Republic of Korea from November 2010 to January 2012 were reviewed. Criteria for eligibility included patients less than 80 years of age diagnosed with LSS with at least a 3-month history of LSS symptoms and neurological claudication. All patients were required to meet the radiological diagnostic criteria, including an anterior-posterior (AP) diameter of the spinal canal less than 12-mm in MRI axial images [1].

**Figure 1:**
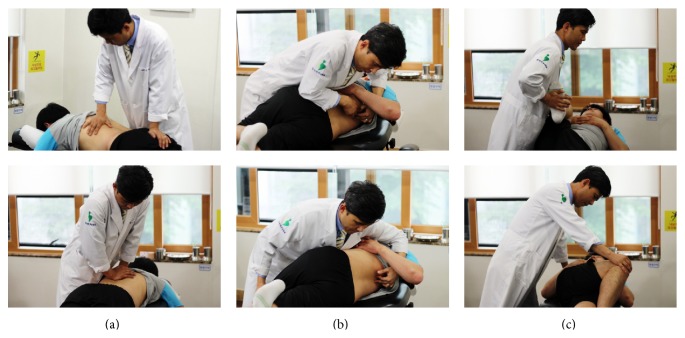
The Chuna procedure (Korean-style manual therapy). (a) Extension-mobilization technique of lumbar vertebrae. (b) Manipulation technique of lumbar vertebrae in the lateral recumbent position. (c) Relaxation technique for lumbar vertebrae and the hip joints.

All patients received the same treatment interventions during the admission period; the treatments consisted of body acupuncture, pharmacoacupuncture, Chuna (Korean-style manual therapy), and the oral administration of herbal medicines. Semi-individualized acupuncture points were selected for body acupuncture. GV3, GV4, BL23, BL25, and GB30 were treated in all patients, and BL56, BL57, GB34, GB39, SP9, and SP6 were selectively treated based on the symptoms of the individual patients. A 0.25∗40-mm disposable stainless steel acupuncture needle (Dong Bang Co., Korea) was used for needling, and acupuncture treatment was conducted once a day. The needle retention time was approximately 15 minutes. Hwangryunhaedoktang pharmacoacupuncture solution (1-2 cc; Korean Pharmacoacupuncture Institute, Korea), which consisted of* Coptidis rhizoma, Scutellariae radix, Phellodendri cortex*,*  *and* Gardeniae fructus* extracts, was injected subcutaneously in the same locations as the acupuncture needles using a 26-gauge insulin syringe once daily. Chuna is a Korean manipulation technique, which is comprised of mobilization within the limit of the passive range of joint motion and muscle relaxation according to the patient's respiration (Figure 1) [11]. The patients attended Chuna manual therapy five times per week. Herbal decoctions mainly consisting of* Eucommiae cortex, Achyranthis radix, Cibotii rhizoma, Sorbus commixta, Geranium thunbergii, Saposhnikoviae radix*,* and Acanthopanacis cortex* were administered three times a day. In addition to these core treatments, information for daily activities and walking exercise was offered to patients by consultation with a KM doctor five times a week. Only low back and lower limb stretching exercises at the bed side and flatland walking were allowed. It was recommended that patients should not walk past the pain threshold and that they increase walking distance in a stepwise manner. A KM doctor evaluated the condition and exercise status of the patients at every consultation. All treatments were continued for the admission period of approximately 1 month for each patient.

A 0 (no pain) to 10 (worst possible pain) visual analogue scale (VAS) for the daily average of low back pain and the walking duration without pain were used to assess patients before and after admission treatment. The telephone survey of each patient was conducted one year after treatment by the same KM doctor who assessed the VAS score and walking duration without pain. Statistical analyses were conducted with a *t*-test or Wilcoxon signed-rank test based on the normality of data with PASW Statistics 18 software (Polar Engineering and Consulting). The patients were evaluated for adverse events at every treatment. All of the study personnel complied with the ethical guidelines of the Mokhuri Neck and Back Hospital regarding all treatment procedures and patient medical record assessments.

## 3. Results

A total of 39 patients were admitted for treatment of symptoms related to LSS, and 4 patients were excluded from the study because they did not meet the diagnostic criteria for LSS; that is, the AP diameter of the spinal canal was over 12 mm on an L-spine MRI. In addition, 2 patients were excluded from the study during the treatment course; 1 refused admission for treatment, and the other developed an elevated serum aspartate aminotransferase (AST) level. As a result, the medical records of 33 patients were reviewed. Among the 33 LSS patients, 24 were contacted by telephone and finished the secondary interviews.

A total of 5 male and 28 female patients received the treatments. The median age was 66 years old (25% and 75% quartile ranges of 62 and 73 years, resp.), and the average disease duration was 9 (SD: 9.42) months. The average admission period was 28.75 (4.11) days. Seven patients were advised to undergo decompression surgery, and 13 had previously undergone a nerve block treatment. The L4 to L5 intervertebral space was the most frequent location where stenosis occurred (26 patients) followed by the L3 to L4 space (10 patients), the L2 to L3 space (8 patients), and the L5 to S1 space (4 patients); 13 patients had multilevel lesions. According to the radiological diagnostic criteria, absolute LSS (an AP diameter of the spinal canal of <10 mm) was observed in 14 patients, and the remaining patients showed relative LSS (an AP diameter 10 to 12 mm). Four patients were diagnosed with spondylolysis, and 9 were diagnosed with spondylolisthesis.

**Figure 2:**
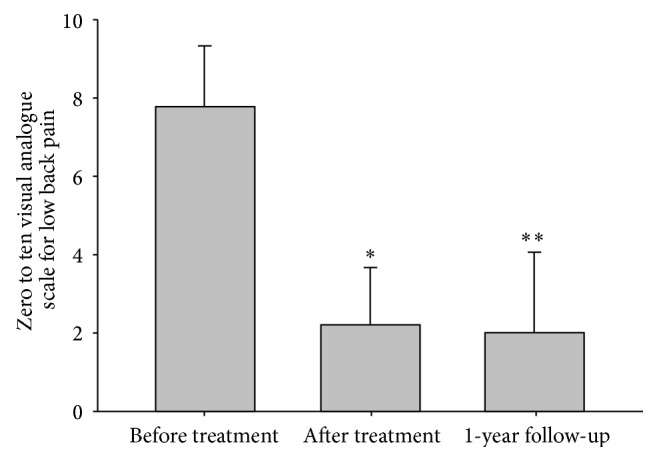
Visual analogue scale of pain. ∗: Wilcoxon signed-rank test of the before and after treatment values, *p* < 0.05; ∗∗: *t*-test of the before treatment and after one year of follow-up values, *p* < 0.05.

**Figure 3:**
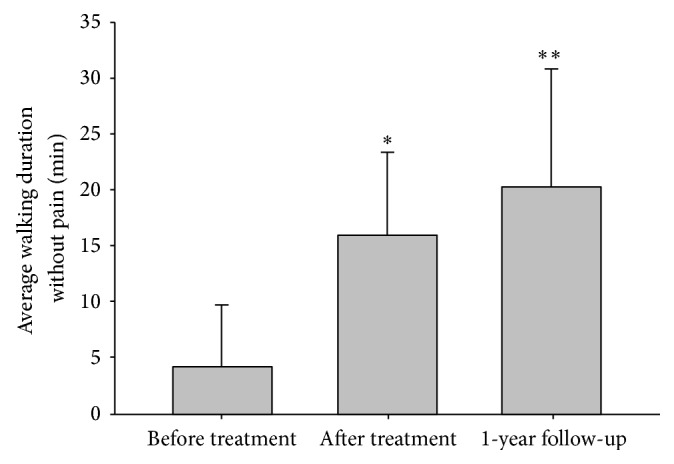
Walking duration without pain. ∗: Wilcoxon signed-rank test of the before and after treatment values, *p* < 0.05; ∗∗: *t*-test of the before treatment and after one year of follow-up values, *p* < 0.05.

Not all of the outcome data were normally distributed according to the Shapiro-Wilk test for normality. Therefore, the Wilcoxon signed-rank test was used for the statistical analysis on the difference between before and after treatments. The average VAS score for pain was significantly decreased from 9 (1.15) to 2.75 (2.22) (*p* < 0.01, Figure 2). The average walking duration without pain was also significantly improved from 5.5 (6.66) to 16.75 (13.00) minutes (*p* < 0.01, Figure 3). No adverse events were reported during the treatment period.

To assess the midterm effects of KM treatment for LSS, data from the 24 patients who finished the one-year follow-up telephone interview were used for analysis. Among the 24 patients, no patient underwent spinal surgery during the follow-up period. Statistical analyses were conducted with a *t*-test based on the normality of data. The pain VAS score decreased significantly during the treatment from 7.88 (1.48) to 2.33 (1.37, *p* < 0.001), and it continued to improve over the one-year follow-up period (2.10 (1.98), *p* < 0.001, Figure 2). The walking duration without pain also showed significant improvement during the treatment (4.21 minutes (5.48) at the pretreatment and 16.00 (7.30) minutes at the posttreatment assessments), and it continued to improve after one year (20.25 (10.56), *p* < 0.001, Figure 3).

## 4. Discussion

The results of this study demonstrate that approximately one month of nonoperative KM combination therapy may result in decreased pain and improved function with a comparatively low adverse event rate for LSS patients. The patients had extremely severe pain (average VAS: 9) at the beginning of the study, but only mild pain remained shortly after the treatment (average VAS: 2.75). Considering that the minimum clinically important difference in low back pain after spinal fusion surgery is approximately 2.2 points based on the recent literature [12], a 6.25-point decrease for a 0 to 10 VAS for back pain indicates considerable clinical significance. Neurological claudication was also significantly improved. Patients could walk without leg pain for more than three times as long after treatment (from 5.5 to 16.75 minutes). No participant complained of adverse events related to the treatments. In addition, the study also showed that the decreased pain level and improved function continued for one year. Among the 24 patients who participated in the telephone survey, the severity of low back pain and walking duration without pain continued to improve after one year of follow-up.

The possible mechanism of KM combination interventions for the treatment of LSS has not been previously well established. However, a mechanism can be hypothesized based on the effects of separate individual interventions, which have been assessed in previous studies. Evidence of the effectiveness of acupuncture for low back pain has been shown, and practitioners generally agree with its clinical efficacy [13]. A previous animal experimental study suggested that acupuncture at the lumbar region may enhance sciatic nerve blood flow as well as the peripheral vasa nervorum through regulation of blood pressure and vasodilator nerve activity, which comprises a possible mechanism for the improvement of claudication [14]. The herbs used in this study might regulate inflammatory mediators and affect the chronic inflammatory conditions of the spinal lesion [15–17]. Although Chuna manual therapy has not been shown to be as effective on LSS as chiropractic or other types of manipulation techniques [18], it might relax the shortened muscles and correct the deformity related to long-term changes in the spinal bony structures and their arrangement, contributing to the functional improvement of LSS. Hwangryunhaedoktang pharmacoacupuncture is a novel Korean acupuncture technique that is believed to simultaneously deliver the effects of herbal medicine and acupuncture-point stimulation. Currently, clinical evidence has not been established for this treatment, but previous experimental studies suggest that it might have anti-inflammatory and analgesic effects in animal models [19]. The individual effects of each intervention may combine and contribute to the positive clinical result as a whole.

This study has several limitations. First, as we mentioned earlier, conclusive evidence for the use of combination therapy to treat LSS cannot be established from this study because case series have only limited value and occupy a low grade in the hierarchy of levels of evidence. Second, only short-term effects were evaluated, and well-validated, standardized, LSS-specific outcome assessment tools were not used. In particular, walking capacity was needed to be evaluated using condition-specific tools or questionnaires with ensured validity and credibility to support the clinical evidence [20]. The evaluation of walking duration without pain is a well-tolerated and easy clinical outcome for walking capacity in LSS patients, but the validity and credibility were not tested. In future clinical trials, appropriate outcomes for pain and function of LSS patients should be adopted. Third, in the follow-up telephone survey, we could not contact all of the patients who were included in the medical record review. This might exaggerate treatment effects and emphasize the positive results after one year. Fourth, due to the methodological limitations of this case series, we cannot confirm whether each treatment was effective individually or if a combination of the various treatments was a key factor for the clinical improvements seen in patients. Future clinical trials comparing the clinical effectiveness of individual interventions will be necessary for the assessment of individual and combined effects. Finally, the relevance of the study results cannot be ensured because the study results only reflect our specific experience with Korean patients with respect to LSS treatment. Various interventions are usually applied to treat low back pain in Korean medicine, which are not commonly used in biomedical clinics. A recent survey of Korean medicine (KM) suggests that general Korean medicine hospitals offer combination treatments with herbal medication, acupuncture, pharmacoacupuncture, physiotherapy, and Chuna to most patients with low back pain simultaneously [21]. Additionally, comparatively long-term nonsurgical, inpatient, and alternative therapies are quite common in Korea. In this sense, the clinical outcomes should be expected to be more conservative in a different cultural context.

Despite these limitations, this study is, to our knowledge, the first English language report of KM treatment for LSS. We hope the clinical efficacy and effectiveness of KM combination treatment for LSS will be established through future rigorous RCTs with larger sample sizes. A standardized treatment protocol for KM treatment using the appropriate assessment tools will be necessary for future studies.
